# Retrieval of Separated Intracanal Endodontic Instruments: A Series of Four Case Reports

**DOI:** 10.7759/cureus.35694

**Published:** 2023-03-02

**Authors:** Ajit Hindlekar, Gurmeen Kaur, Rasika Kashikar, Pratik Kotadia

**Affiliations:** 1 Conservative Dentistry and Endodontics, Dr. D. Y. Patil Dental College and Hospital, Dr. D. Y. Patil Vidyapeeth, Pune, Pune, IND; 2 Conservative Dentistry and Endodontics, Sinhagad Dental College and Hospital, Pune, Pune, IND; 3 Endodontics, Private Practitioner, Pune, IND

**Keywords:** case report, ultrasonics, separated instrument, instrument retrieval, root canal complication

## Abstract

The separation of an endodontic instrument during a root canal procedure is one of the most common endodontic mishaps. Separation of endodontic instruments may block access to the apical portion of the root and hamper the disinfection process. It obstructs the appropriate debridement of the canal apical to the fragment, jeopardizing the treatment's outcome. However, due to the advancements in the methods and armamentarium, the effective retrieval of a separated instrument (SI) from the root canal has become possible. This paper includes a case series of management of separated instruments whereby SI was successfully removed in four cases. The instruments were separated intracanal at various levels in the middle and apical third of maxillary and mandibular molar teeth. The level of separation was located, staging performed, and SI was removed using an ultrasonic device under magnification. Removal of the SI was followed by obturation till the entire working length and subsequent post-endodontic restoration. Patient satisfaction with treatment outcomes in all cases was good. Case evaluation, good armamentarium, adequate knowledge along with good clinical skills and experience aid in the successful retrieval of separated instruments. Removal of the instrument without further damage to radicular dentin is important to maintain the integrity of the tooth.

## Introduction

According to the American Association of Endodontics, each year, more than 15 million teeth undergo endodontic treatment [1]. During routine therapy, at any stage, an endodontic instrument can fracture due to flexural fatigue, torsion or manufacturing defects [2]. The incidence rate of intracanal fracture of endodontic hand instruments ranges between 0.25% and 6% [3]. For treating infected root canals, the removal of infected pulp tissue and obstacles is important for efficient disinfection and shaping of the canals. A separated instrument (SI) poses major challenges, including the possibility of intracanal corrosion and limited or no accessibility for chemo-mechanical preparation of the canal apical to the SI. Hence, removal of separated instrument or bypassing is necessary to gain access to the entire length of the canal [2]. The removal of SI depends upon the canal configuration, type of instrument, and its location in the canal. The presence of a gap between the SI and root canal walls eases the removal process. Removal of an SI is more predictable in maxillary teeth and anterior teeth than in mandibular teeth and posterior teeth [4]. Moreover, a fractured instrument fragment located in the coronal third of the root has a better prognosis because of improved visibility, accessibility, and conservation of radicular dentin during the retrieval procedure [3]. Hand instruments like Hedstroem files are more difficult to remove than K-files due to deeper engagement in dentin [5].

Larger rakes and helix angles and deeper flutes in the file increase the difficulty of retrieval [6]. Rotary files, because of their tendency to thread into root canal walls, are more difficult to remove than hand files [7]. While removal, the vibrations generated by ultrasonics may shorten the fragment or separate it further. Owing to the shape memory of NiTi, the separated fragment may straighten and reengage into the dentin [8]. The clinician’s persistence, methodologic approach, and familiarity with ever-changing technology are essential for the successful management of SIs. An experienced clinician retrieves the SI without unnecessary removal of tooth structure [9]. If a clinician does not feel capable of retrieving an SI in a particular case, then immediate referral is necessary to avoid risking further compromise of the tooth. Before finalizing any retreatment plan, the clinician must inform the patient about all viable treatment options and their associated risks and benefits. Once the patient agrees to a treatment plan, the clinician must obtain written informed consent [10]. The paper contains a few case reports pertaining to strategies for the management of a SI.

## Case presentation

Case 1

**Figure 1:**
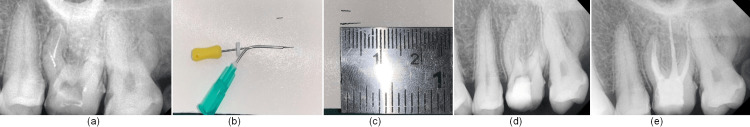
SI removal: (a) intraoral periapical showing two SI in mesiobuccal canal of maxillary first molar. (b) 22-gauge (0.7-mm-wide) hypodermic needle with a No. 20 H-file for removal of SI. (c) Two SI retrieved. (d) Intraoral periapical showing a clear mesiobuccal canal. (e) Intraoral periapical showing obturated maxillary first molar.

A 33-year-old male patient, referred by a general dentist, complained of mild, intermittent pain for two weeks which aggravated on mastication. The patient reported a history of dental treatment initiated one month earlier. On intraoral examination, a large occlusal cavity in the right maxillary first molar (16) was observed, which was tender on percussion. An Intraoral Periapical (IOPA) radiograph of the tooth revealed two SI in the mesiobuccal canal and substantial periapical pathosis (Figure 1a). Based on the clinical and radiographic analysis, a diagnosis of Previously Initiated Therapy was reached upon. Retreatment was initiated under rubber dam isolation. An ultrasonic tip (ProUltra ultrasonic tip No. 2) was used to achieve straight-line access to the SI so that the coronal portion of the SI could be visualized through a dental operating microscope (ProErgo, Carl Zeiss Meditec). An ultrasonic tip No. 2 was used to remove the first SI and a 22-gauge (0.7-mm-wide) hypodermic needle with a No. 20 H-file was used to remove the second SI (Figure 1b). The needle was modified by flattening its tip and roughening its smooth lumen with a small, tapered fissure bur. The modified needle was rotated counterclockwise under minimum apical pressure to cut a groove around the coronal end of the SI. Once the needle was secured around the SI, the H-file was wedged carefully between the SI and the needle’s inner wall in a clockwise turning motion. The entire assembly (needle, H-file, and SI) was pulled out slowly in a rotational movement. After retrieving the SI (Figure 1c), the canals were thoroughly debrided, and an intracanal calcium hydroxide paste was placed. The patient was then transferred back to the general dentist, who reported that the tooth was asymptomatic and obturation was completed followed by a post-endodontic restoration.

Case 2

**Figure 2:**
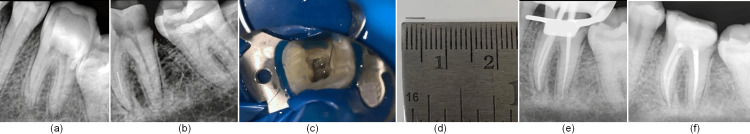
SI removal: (a) intraoral periapical showing deep restoration involving the pulp chamber. (b) Separated instrument in mesiolingual canal of mandibular first molar. (c) Retrieved instrument fragment seen clinically. (d) Measurement of retrieved SI. (e) Master cones in mesiobuccal, mesiolingual, and distal canals. (f) Intraoral periapical showing obturated mandibular molar.

A 38-year-old female patient was referred with the chief complaint of pain in the lower left back tooth region for the past one month, and she gave a history of restoration done around a year back. On intraoral examination, dislodged restoration was observed in the left mandibular first molar (36) which was hyper-responsive to cold test with lingering pain and positive response to percussion. A diagnostic radiograph revealed a deep restoration involving the pulp chamber (Figure 2a). Based on the clinical, radiographic examination and vitality tests, a diagnosis of symptomatic irreversible pulpitis was made. Hence non-surgical endodontic therapy was planned to treat the involved teeth. After rubber dam isolation, the restoration was removed, and the access cavity was prepared followed by working length determination. The root canals were prepared till F3 using ProTaper Universal rotary file system (Dentsply Maillefer, Ballaigues, Switzerland). However, while cleaning and shaping the canals, the ProTaper F3 file got separated in the mesiolingual canal around the middle third (Figure 2b). Retrieval of the instrument was planned wherein the radicular access to the coronal end of the SI was straightened by sequential use of modified GG drills. Acteon Satelec P5 neutron ultrasonic generator with ultrasonic tips ET25 (Satelec Acteon, France) was activated at a power setting 6 to trephine dentin around the broken fragment. The procedure was carried out under magnifying loupes (×2.5 magnification) (Heine, Germany). Canal was irrigated with normal saline intermittently to flush out the debris from the canal and act as a coolant. After about 30 min, the fragment loosened and popped out of the canal (Figure 2c) which was measured to be around 6mm (Figure 2d). IOPA radiograph confirmed the removal of the SI. Working length was then determined, followed by cleaning and shaping of the root canal system with ProTaper Universal rotary system and single cone obturation using SealApex sealer (Figure 2e). Following obturation, the tooth was restored with resin composite restoration (Filtek Z350 XT Universal Restorative, 3M India) (Figure 2f).

Case 3

**Figure 3:**
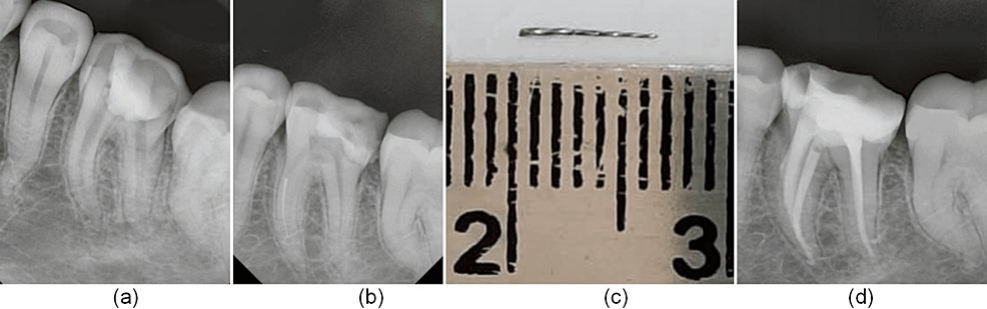
SI removal: (a) intraoral periapical showing deep restoration involving the pulp chamber. (b) Separated instrument in mesiobuccal canal of mandibular first molar. (c) Retrieved instrument fragment measured. (d) Intraoral periapical showing obturated mandibular molar.

A 22-year-old male patient reported with the chief complaint of pain in the lower right back tooth region in the last 1 month. The pain was mild and intermittent in nature, which aggravated mastication. He gave a history of incomplete root canal treatment in the right mandibular first molar and experienced pain a few days after undergoing endodontic therapy (incomplete). Intraoral examination revealed a temporary/provisional restoration with tooth #46 with no other detectable abnormality. The involved tooth was severely tender on percussion. Radiographic examination revealed a deep restoration involving the pulp chamber (Figure 3a). Based on the history and the clinical and radiographic examination, a diagnosis of Symptomatic apical periodontitis was made and according to AAE, 2013 this was categorized under “Previously Initiated Therapy.” The completion of the root canal treatment was planned. After isolation with a rubber dam, the temporary restorative material was removed and the access opening was refined. Three canal orifices were located, and the working length was determined. However, during cleaning and shaping, an F2 Protaper Universal file separated in a mesiobuccal canal in 6. A radiograph was taken to confirm the level of separation of the instrument (Figure 3b). The instrument was found to be separated at the junction of the coronal and middle third of the root canal. A staging platform was created using a Gates Glidden bur (size #2; Dentsply-Maillefer) to expose the tip of the separated fragment in the MB canal, and an ultrasonic tip (Satelec/Acteon, Merignac, France) was then used intermittently at a medium frequency (36 kHz) without simultaneous coolant irrigation to successfully retrieve the instrument fragment (Figure 3c). The working length was determined radiographically, and the canal was prepared using the Protaper Universal system (Dentsply Maillefer) to size F3, with copious irrigation of 5% NaOCl solution followed by 17% EDTA. The canals were dried and obturated using single cones and AH-plus sealer (Dentsply Detrey Gmbh, Konstanz, Germany) (Figure 3d). The access cavity was immediately sealed with glass ionomer restorative material and then replaced one week later by a composite restoration (GC America, Chicago, IL, USA).

Case 4

A 27-year-old male reported with severe, continuous pain in the mandibular left back teeth region for one week. The patient gave a history of endodontic treatment one month back, but the pain was not alleviated. Clinically, a large cavity restored with composite was observed in the mandibular left first molar (36). An IOPA showed an under-obturated distal root canal with a periapical radiolucency and an SI in the middle third of a mesiolingual canal. Since the patient was symptomatic, retreatment was planned, and the procedure was carried out under the dental operating microscope. Under rubber dam isolation, the attempt to retrieve SI was made. A number 8 K-file (Dentsply Sirona) without the use of any chelating agent was first used to bypass the SI. Several radiographs were obtained throughout the procedure to avoid any complications, such as ledge formation, secondary instrument fracture, apical extrusion of the SI, or root perforation. Once the fragment was bypassed with the smaller file, sequential instrumentation with K-files (up to No. 25) was performed. Using copious irrigation, two unused Hedstrom files of size 20 and 25 were used to bypass the SI, and engaged as deep as possible, twisted clockwise using the file braiding technique. Braiding of these files and a short outward pull resulted in the instrument being removed from the canal. Next, the gutta-percha removal was done from the distal canal using ProTaper Retreatment instruments (D1-D3, Dentsply Tulsa Dental) at approximately 500-750 RPM. Chemomechanical preparation was completed for all three canals using hand files followed by Protaper Universal rotary till F2. An intracanal dressing of calcium hydroxide paste was then applied and was recalled after two weeks. After all the symptoms had subsided, obturation and post-obturation composite restoration were completed. At a subsequent visit, two weeks later, the tooth was restored with a porcelain-fused-to-metal crown. The patient was satisfied with the treatment outcome.

**Figure 4:**
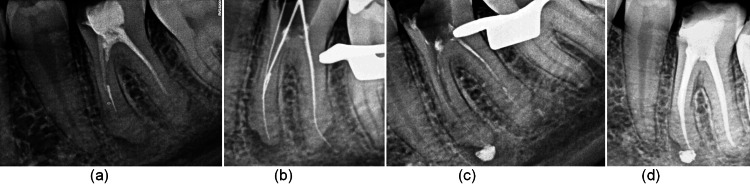
SI removal: (a) intraoral periapical showing previously treated with a separated instrument in mesiobuccal canal of mandibular first molar. (b) SI bypassed with size 8 K-file. (c) Instrument fragment retrieved and calcium hydroxide intracanal medicament removed after two weeks. (d) Intraoral periapical showing obturated mandibular molar.

## Discussion

Instrument fracture is more frequently reported in molars, with higher occurrence in the mesiobuccal roots [11]. Management of a separated instrument can be done by bypassing it and reaching the full length of the canal; or cleaning, shaping, and obturating the canal till the coronal point of separation [12]. With the recent techniques of instrument retrieval, instrument removal has become more predictable [13].

For removing SI from the root canal, several techniques have been reported, including the Masserann kit, Endo Extractor, wire loop technique, and ultrasonics [14]. The SI was cited at distinct levels in this report, all of which were successfully eliminated. The whole process is better guided by the use of a microscope or magnifying loupes, which minimizes injury to the canal dentine. The position of the instrument in relation to the canal curvature, the depth of the instrument within the canal, the type of separated instrument and the size of the fragment all play a role in the successful removal of separated instruments. If an instrument is lying in the straightaway area of the canal and one-third of its whole length is exposed, it can be easily retrieved [15,16]. According to Nevares et al., the success rate of retrieval was 85.5% when the separated fragment was visible using a dental microscope, compared to 47.7% when the fragment was not visible [12,15]. It is frequently impossible to retrieve a broken instrument that is apical to canal curvature. Stainless steel files are easier to remove than NiTi files, which have a tendency to break more during retrieval due to heat accumulation [16]. Ultrasonics was first introduced in endodontics in 1957. Initially, ultrasonic units were used with frequencies between 25-40 kHz, but recently handpieces operating at 1-8 kHz have been introduced that cause low shear stresses and lesser changes in canal surface [17]. The SI is removed in these cases using the Aceton Satelec P5 neutron, a piezoelectric ultrasonic generator. These units' tips move back and forth in a linear, "piston-like" manner, which is suitable for endodontics [5,17]. The heat generated due to friction between ultrasonic tips and canal dentin can cause instrument fatigue and secondary fracture of the separated fragment. Hence, ultrasonic tips at low power levels are employed for shorter application periods [18]. to obtain unobstructed visibility under DOM, the ultrasonic tip should be activated in a dry field without the simultaneous use of coolant irrigant, the power should be set at medium, and the device should be applied intermittently [19].

Although there is not much difference between the endodontic outcomes for teeth with retained or retrieved separated fragments, the prognosis may be affected by the preoperative pulpal and periodontal status [20]. The removal of separated instrument fragments without further damaging the radicular dentin is of utmost importance. Hence, the preferable technique for the successful removal of SI would be the use of ultrasonic tips in combination with DOM after creating a staging platform.

## Conclusions

The decision on the superiority of an instrument retrieval method is complex. So before attempting to remove the instrument, a thorough examination of the situation and consideration of the potential risks should be considered. The shape of the root canal, the state of the tooth structure's restorative work, the availability of the armamentarium, and the case's prognosis should all be taken into account. For a successful outcome, each case must be evaluated and planned individually, and care must be given throughout any retrieval attempt to minimize further canal damage.
